# Cerebrospinal fluid neurogranin and TREM2 in Huntington’s disease

**DOI:** 10.1038/s41598-018-21788-x

**Published:** 2018-03-09

**Authors:** Lauren M. Byrne, Filipe B. Rodrigues, Eileanoir B. Johnson, Enrico De Vita, Kaj Blennow, Rachael Scahill, Henrik Zetterberg, Amanda Heslegrave, Edward J. Wild

**Affiliations:** 1 Institute of Neurology, University College London, Queen Square, London, WC1N 3BG UK; 2 Clinical Neurochemistry Laboratory, Sahlgrenska University Hospital, S-431 80 Mölndal, Sweden; 3 Department of Psychiatry and Neurochemistry, the Sahlgrenska Academy at the University of Gothenburg, S-431 80 Mölndal, Sweden; 4 UK Dementia Research Institute at UCL, London, WC1N 3BG UK

## Abstract

Biomarkers of Huntington’s disease (HD) in cerebrospinal fluid (CSF) could be of value in elucidating the biology of this genetic neurodegenerative disease, as well as in the development of novel therapeutics. Deranged synaptic and immune function have been reported in HD, and concentrations of the synaptic protein neurogranin and the microglial protein TREM2 are increased in other neurodegenerative diseases. We therefore used ELISAs to quantify neurogranin and TREM2 in CSF samples from HD mutation carriers and controls. CSF neurogranin concentration was not significantly altered in HD compared to controls, nor was it significantly associated with disease burden score, total functional capacity or motor score. An apparent increase in CSF TREM2 in manifest HD was determined to be due to increasing TREM2 with age. After age adjustment, there was no significant alteration of TREM2 in either HD group, nor any association with motor, functional or cognitive score, or brain volume quantified by MRI. Both analyses were well-powered, and sample size calculations indicated that several thousand samples per group would be needed to prove that disease-associated alterations do in fact exist. We conclude that neither neurogranin nor TREM2 is a useful biofluid biomarker for disease processes in Huntington’s disease.

## Introduction

Huntington’s disease (HD) is an autosomal dominant neurodegenerative disease caused by CAG repeat expansions in *HTT* encoding mutant huntingtin protein^1^. The pathogenesis of HD is multifactorial and includes synaptic dysfunction^2^ and activation of the innate immune system, most likely due to a direct effect of mutant huntingtin in myeloid cells^3–5^. We previously showed that cytokines^3,4^ and chemokines^6^ are increased in plasma in HD mutation carriers and that CSF in HD contains increased levels of the microglia-associated proteins chitotriosidase and YKL40, with the latter independently associated with the severity of motor symptoms^7^. Modulating the immune system has the potential to offer therapeutic benefit in HD^8^ and one trial of a putative microglial-modulating agent (laquinimod) is currently underway^9,10^.

Neurogranin is a postsynaptic protein that regulates the availability of calmodulin^11^ that has been proposed as a synaptic function biomarker^12^. Neurogranin has been shown to be increased in CSF in Alzheimer’s disease (AD)^13^ but not in other neurodegenerative conditions such as frontotemporal dementia (FTD), Lewy body disease, Parkinson’s disease (PD), progressive supranuclear palsy and multiple system atrophy^14^. There is evidence that synaptic dysfunction contributes to HD pathology^15,16^, and a whole-brain gene expression study in post-mortem HD patient brains identified that *NRGN*, encoding neurogranin, was among the most robustly downregulated genes in HD caudate compared to controls^17,18^. However, it has not previously been quantified in CSF in HD.

Triggering receptor expressed on myeloid cells-2 (TREM2) is a cell surface receptor expressed by myeloid cells including monocytes, macrophages and microglia whose activation is inhibitory to the immune response^19^. Missense mutations in *TREM2* are associated with CNS disease^20^ and single-nucleotide *TREM2* polymorphisms have been reported as genetic modifiers of AD^21^, amyotrophic lateral sclerosis^22^, PD and FTD^23^. Soluble TREM2 is quantifiable in CSF and has been reported as elevated in AD^24,25^, and in multiple sclerosis, where it normalised upon immunomodulatory treatment^26^. While TREM2 has not specifically been linked to the pathobiology of HD, dysfunction of myeloid cells due to cell-autonomous expression of mutant huntingtin is a well-described feature of the disease^5^, and other microglial-associated proteins have shown disease-related alterations in HD patient CSF^7^. Our previous work demonstrates the principle that biomarker studies in human biofluids can provide novel pathogenic insights by highlighting links with substances previously reported to be linked to HD^3,27,28,29^.

On the basis of these findings in other neurological conditions and the potential to show alteration in CSF in HD, we therefore set out to quantify neurogranin and soluble TREM2 in CSF samples from HD mutation carriers and matched controls.

## Results

### Neurogranin

The neurogranin cohort consisted of 32 participants: 12 healthy controls and 20 HD gene expansion carriers. The HD group contained 17 manifest and 3 premanifest HD participants pooled together. Details are given in Table 1. There was no significant difference in age (p = 0.243) or gender (p = 0.452) distribution between the two groups.

**Table 1:** Characteristics of the neurogranin cohort (values are median (interquartile range)) and CSF neurogranin concentrations (values are median (interquartile range; mininum - maximum)). HD, HD gene expansion carriers; CAG, CAG triplet repeat count; DBS, disease burden score.

Group (n)	Control (12)	HD (20)
Age	40 (25)	54 (13)
Sex F/M	3/9	9/11
CAG	N/A	44 (4)
DBS	N/A	401 (127)
Total functional capacity	N/A	11 (3)
UHDRS Total motor score	N/A	26 (24)
Neurogranin (pg/mL)	40.5 (100.5; 10–282)	43.5 (72.5; 10–392)

**Figure 1:**
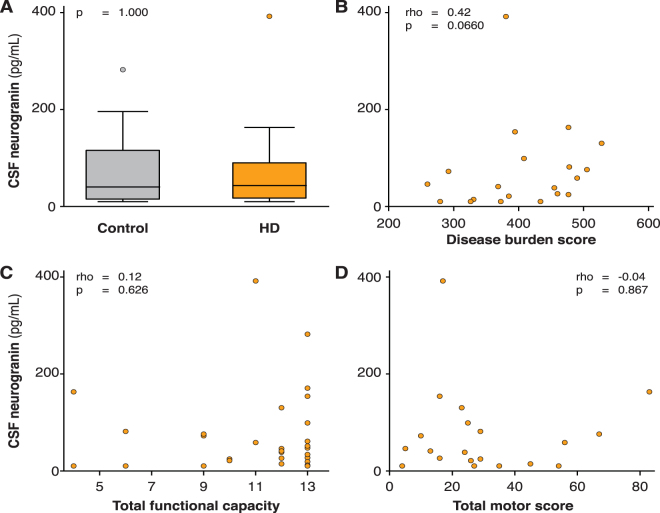
No evidence for altered CSF neurogranin levels in HD. (**A**) Inter-group comparison between controls (n = 12) and HD gene expansion carriers (n = 20). (**B**–**D**) Relationship between CSF neurogranin concentration and (**B**) disease burden score; (**C**) UHDRS total functional capacity score and (**D**) UHDRS total motor score.

Medians and interquartile ranges (IQR) of CSF neurogranin are shown in Table 1. CSF neurogranin concentration did not vary between genders (p = 0.984), or with age in healthy controls (rho = −0.09, p = 0.787). The concentration of CSF haemoglobin was not associated with the concentration of CSF neurogranin (rho = −0.25, p = 0.585). CSF neurogranin concentration was not significantly different between healthy controls and HD (Fig. 1; p = 1.000). There was no significant correlation between CSF neurogranin levels and disease burden score (rho = 0.42, p = 0.066), UHDRS total functional capacity score (rho = 0.12, p = 0.626) or UHDRS total motor score (rho = −0.04, p = 0.867).

**Table 2:** Characteristics of the TREM2 cohort (values are mean ± SD) and CSF TREM2 concentrations (mean ± SD of square-root transformed values). CAG, CAG triplet repeat count; DBS, disease burden score; TFC, total functional capacity; TMS, total motor score.

Group (n)	Control (20)	Premanifest HD (20)	Manifest HD (40)
Age	50.7 ± 11.0	42.4 ± 11.0	56.0 ± 9.37
Sex F/M	10/10	10/10	18/22
CAG	N/A	42.0 ± 1.62	42.8 ± 2.18
Disease burden score	N/A	267.1 ± 61.9	395.3 ± 94.6
Total functional capacity	13 ± 0	13 ± 0	9.4 ± 2.70
Total motor score	2.35 ± 2.43	2.80 ± 2.80	37.3 ± 19.3
CSF TREM2 concentration (√pg/mL)	77.5 ± 12.5	75.4 ± 11.6	87.6 ± 16.7

A post-hoc power calculation showed that this dataset had 98% power to determine that CSF neurogranin levels are equivalent between healthy controls and HD gene expansion carriers. A sample size calculation indicated that 14,661 samples per group would be needed to establish with 80% power at p = 0.05 that neurogranin levels are in fact higher in HD than in controls. Therefore the analysis was not repeated in the larger TREM2 cohort.

### TREM2

The TREM2 cohort consisted of 80 independent CSF samples collected from 20 healthy controls, 20 premanifest *HTT* mutation carriers, and 40 patients with manifest HD, stages 1–3. Demographics and clinical characteristics are given in Table 2. The premanifest HD group was significantly younger than the control and manifest HD groups (ANOVA p < 0.0001; control versus premanifest HD, p = 0.012; premanifest versus manifest HD p < 0.0001; p = 0.0244 and p =< 0.0001 after Bonferroni correction for 2 comparisons), emphasising the need to adjust analyses for age, but there were no inter-group differences in gender (p = 0.905).

**Figure 2:**
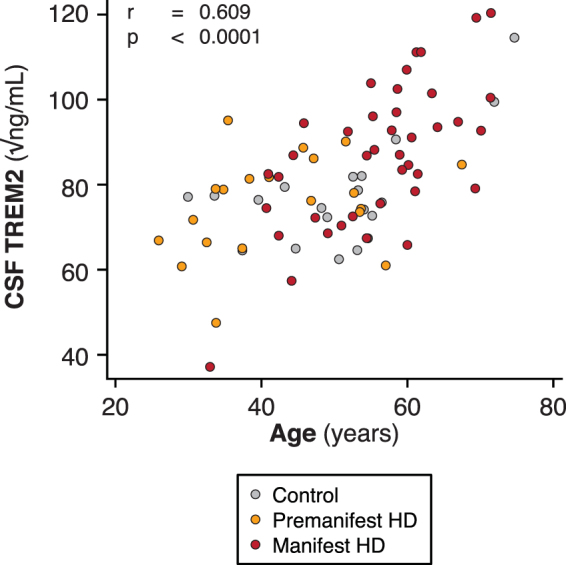
Relationship between CSF TREM2 and age in controls and HD gene expansion carriers.

**Figure 3:**
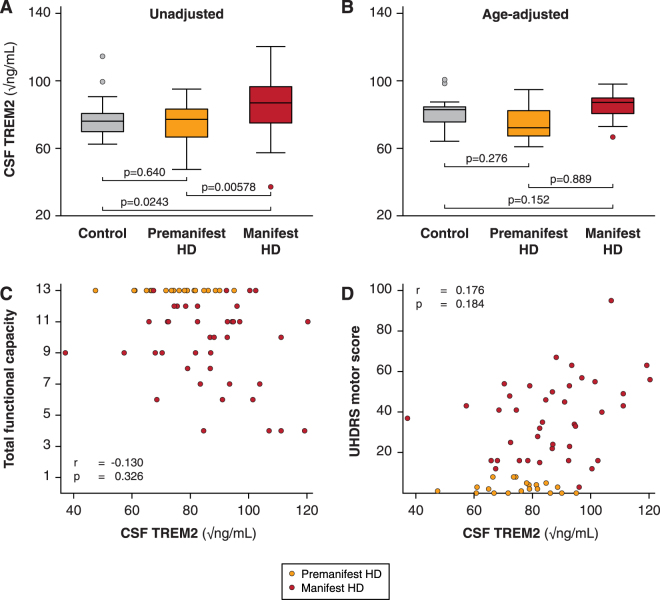
CSF TREM2 is not associated with disease stage or other characteristics in HD. (**A**) CSF TREM2 in controls, premanifest HD and manifest HD before age-adjustment. (**B**) Same data after age-adjustment. Relationship between CSF TREM2 and (**C**) UHDRS total functional capacity and (**D**) UHDRS total motor score. (**C**–**D**) Show raw values in the scatter plot but r and p values are after age adjustment. For relationships with other clinical variables, see text and Table 3.

CSF TREM2 concentrations were strongly associated with age overall (Fig. 2; r = 0.609, p < 0.0001) as well as within the control and HD mutation carrier groups (r = 0.625, p = 0.00320 for control; r = 0.610, p < 0.0001 for HD), so subsequent analyses included age as a covariate. There was no evidence for an effect of gender on TREM2 concentration in controls or HD gene expansion carriers (p = 0.403 and 0.808 respectively). The concentration of CSF haemoglobin, used to evaluate any effect of blood contamination, was not significantly associated with the concentration of CSF TREM2 (p = 0.741). With age as a covariate, TREM2 concentration was not significantly different in HD gene expansion carriers overall than in controls (77.5 v 83.4 √pg/mL, p = 0.133). In the unadjusted dataset, significant TREM2 increases in manifest HD compared to premanifest HD (p = 0.00578) and controls (p = 0.0243) were seen (Fig. 3A). However, with age included as a covariate, the differences were no longer significant (p = 0.152 and p = 0.889 respectively), suggesting the finding was an artefact of the tendency of TREM2 to increase with age, combined with the older age of the manifest HD group (Fig. 3B). Predictably, these comparisons remained non-significant after Bonferroni multiplicity correction (p = 0.456 and p = 1.000, respectively). CSF TREM2 concentrations in premanifest HD gene expansion carriers showed no significant difference from controls, before or after age-adjustment (Fig. 3A,B; raw p = 0.640; age-adjusted p = 0.276; Bonferroni-corrected, age-adjusted p = 0.828). There was no significant association among HD gene expansion carriers between age-adjusted CSF TREM2 concentration and total functional capacity (Fig. 3C), total motor score (Fig. 3D), symbol-digit modalities test, Stroop word reading, categorical verbal fluency, Stroop color naming, and volumes of whole brain, caudate, grey matter, white matter, and lateral ventricles (Table 3).

**Table 3:** Relationships between CSF TREM2 concentration and clinical, cognitive and MRI brain volume measures after adjustment for age. All volumetric measures were calculated as a percentage of total intracranial volume.

Variable	r	p-value
UHDRS Total functional capacity	−0.130	0.326
UHDRS Total motor score	0.176	0.184
Symbol-digit modality test	−0.030	0.823
Stroop word reading	−0.112	0.398
Stroop color naming	−0.0369	0.782
Categorical verbal fluency test	−0.142	0.285
Volume of whole brain	0.0656	0.658
Volume of caudate	−0.0042	0.978
Volume of grey matter	0.0235	0.874
Volume of white matter	−0.0830	0.575
Volume of lateral ventricles	0.230	0.116

This sample had 97% power to demonstrate that CSF TREM2 levels are equivalent between healthy controls and HD mutation carriers after age-adjustment. A sample size calculation indicated that 3,288 samples per group would be needed to establish with 80% power at p = 0.05 that a significant CSF TREM2 increase does in fact exist in HD versus controls, after adjustment for age.

## Discussion

In two adequately-powered sample sets, we have shown that neither neurogranin, a putative marker of postsynaptic damage, nor TREM2, a putative marker of microglial function, has an altered concentration in CSF in Huntington’s disease, despite each protein having shown alterations in CSF in other neurodegenerative diseases, and each having potential links to HD pathogenesis. An apparent increase in CSF TREM2 concentration in manifest HD was determined to be an artefact of its tendency to increase with age, rather than a disease effect. We show that the sample sizes necessary to demonstrate alterations of these substances in CSF in HD are likely prohibitively large.

Our results do not negate previously-reported positive findings in regard to neurogranin and TREM2 in other diseases, where both mechanistic links to pathobiology and consistent CSF changes have been shown^13,14,24–26^. Similarly, they do not indicate that these proteins have no role in the pathogenesis of HD, or diminish the importance of synaptic or immune dysfunction as pathogenic pathways; merely that detectable alterations were not found in CSF, a biofluid enriched for brain-derived proteins. Finally, there is still a potential role for either substance as a pharmacodynamic biomarker of target engagement by a compound intended to ameliorate synaptic or microglial dysfunction, since for such markers, a baseline difference from controls is not a prerequisite.

Nonetheless we conclude that neither neurogranin nor TREM2 is likely to be of value as a CSF biomarker for disease processes in Huntington’s disease.

## Methods

### Participants and assessments

Ethical approval was given by the joint University College London/University College London Hospitals ethics committee (neurogranin cohort) and the London - Camberwell St Giles Research Ethics Committee (TREM2 cohort). All patients gave informed written consent before enrolment. All experiments were performed in accordance with relevant guidelines and regulations. For the neurogranin cohort, patient consent, inclusion and exclusion criteria, clinical assessment, CSF collection and storage were as previously published^30^. In brief, samples were collected after an overnight fast at the same time of day and centrifuged and aliquoted rapidly on ice using a standardised protocol and polypropylene plasticware^30^. Healthy controls were contemporaneously recruited, drawn from a population with a similar age to patients, and clinically well, so the risk of incidental neurodegenerative diseases was very low. Relevant aspects of clinical phenotype were quantified using the total functional capacity and total motor score components of the Unified Huntington’s Disease Rating Scale (UHDRS)^31^. Manifest HD as opposed to premanifest HD was defined as UHDRS diagnostic confidence level of 4. Disease burden score, a function of age and CAG repeat length that predicts many features of HD onset and progression, was calculated^32,33^. The TREM2 cohort was drawn from the baseline of a larger, more recent CSF collection initiative entitled HD-CSF. This is a single-site study aligned to the HDClarity study (NCT02855476) with added optional MR imaging and a longitudinal design. Inclusion and exclusion criteria, and methods of CSF collection were virtually identical to the neurogranin cohort; clinical assessment included the measures above plus a cognitive battery consisting of Symbol Digit Modality Test, Categorical Verbal Fluency Test, Stroop Color Naming and Stroop Word Reading (full protocol available at http://hdclarity.net).

### MRI acquisition

For the TREM2 cohort, T1-weighted MRI data were acquired on a 3T Siemens Prisma scanner using a protocol optimised for this study. Images were acquired using a 3D MPRAGE sequence with a TR = 2000ms and TE = 2.05 ms. The protocol had an inversion time of 850 ms, flip angle of 8 degrees, matrix size 256 × 240 mm. 256 coronal slices were collected to cover the entire brain with a slice thickness of 1.0 mm with no gap. Parallel imaging acceleration was used (GRAPPA) and 3D distortion correction was applied to all images.

### MRI Processing

Scans underwent visual quality control prior to processing. No scans were excluded due to the presence of significant motion or other artefacts. Bias correction was performed using the N3 procedure^34^. Volumetric regions of the whole-brain, ventricles and total intracanial volume (TIV) were generated via MIDAS using semi-automated segmentation procedures as previously described^35–37^. SPM12 segment (MATLAB version 2012b) was used to measure the volumes of grey and white matter^38^. Finally, to measure caudate volume the images were processed using MALP-EM^39^, a fully automated software that was recently validated in an HD cohort^40^. All segmentations underwent visual quality control to ensure accurate delineation of the regions. No scans failed processing. Brain volumes are expressed as a percentage of total intracranial volume, to account for overall head size.

### CSF analyte quantification

CSF neurogranin was measured using an in-house ELISA and anti-Ng antibodies NG22 and NG2, essentially as described^41^. All samples but 6 were above the limit of detection LOD (i.e. 10.0 pg/mL) – 4 in the HD group and 2 samples in the control. Samples below the LOD were assigned the LOD concentration (i.e. 10.0 pg/mL). Concentrations of TREM2 were quantified with an in-house Meso Scale Discovery based ELISA, using an adapted protocol from Kleinberger *et al*.^42^. All samples were above the LOD (65.3 pg/mL). Haemoglobin concentration was measured using a commercial ELISA (E88–134, Bethyl Laboratories Inc.) to determine CSF contamination by blood. Quantification for each analyte was run on the same day for all samples using the same batch of reagents. Laboratory operators were blinded to clinical data.

### Statistical analysis

Statistical analysis was performed with Stata 14 software (StataCorp, TX, USA). Significance level was defined as p < 0.05. Both neurogranin and TREM2 concentrations were non-normally distributed; square-root transformation produced an acceptable normal distribution for TREM2, while non-parametric tests were used for neurogranin because of the smaller sample size.

Potentially confounding demographic variables (age, gender) were examined in preliminary analyses and those found significant were included as covariates for subsequent analyses.

For neurogranin, we used two-sample Wilcoxon rank-sum (Mann-Whitney) test or the exact Fisher test to assess intergroup differences of the cohort’s characteristics. Two-group comparisons were tested using Wilcoxon rank-sum (Mann-Whitney) test, and associations were tested using Spearman’s rank-order correlation.

For TREM2, we used unpaired two-samples t-test/ANOVA or the Pearson’s chi-squared test to assess intergroup differences of the cohort's characteristics. Group comparisons were tested using unpaired two-samples t-test/ANOVA for unadjusted comparisons or multiple linear regression for adjusted comparisons. Correlations were tested using Pearson’s correlation and partial correlations for covariate adjustment. Where necessary, multiple comparisons were corrected for using the Bonferroni method.

For both analyses, a post-hoc power calculation for equivalence was computed assuming 1% type I errors^43^ to provide an estimation of the statistical power of the data set to prove robustly that analyte levels in healthy controls and HD mutation carriers are not different. We also performed sample size calculations for theoretical experiments to demonstrate that the level of each analyte was higher in HD mutation carriers than in controls, assuming 5% type I errors and 20% type II errors.

### Disclosures

EJW has participated in scientific advisory boards with Hoffmann-La Roche Ltd, Ionis, Shire, GSK and Wave Life Sciences. All honoraria for these advisory boards were paid through UCL Consultants Ltd, a wholly owned subsidiary of UCL. His host clinical institution, University College London Hospitals NHS Foundation Trust, receives funds as compensation for conducting clinical trials for Ionis Pharmaceuticals, Pfizer and Teva Pharmaceuticals. KB and HZ are co-founders of Brain Biomarker Solutions in Gothenburg AB, a GU Ventures-based platform company at the University of Gothenburg. HZ has served at advisory boards of Eli Lilly, Roche Diagnostics and Pharmasum Therapeutics and has received travel support from TEVA.
